# Supplementary figures and images for: Implementation and evaluation of obstetric early warning systems in tertiary care hospitals in Nigeria
Source: PLOS Glob Public Health. 2022 Jul 20;2(7):e0000225. doi: 10.1371/journal.pgph.0000225 (PMC10022187; doi:10.1371/journal.pgph.0000225)

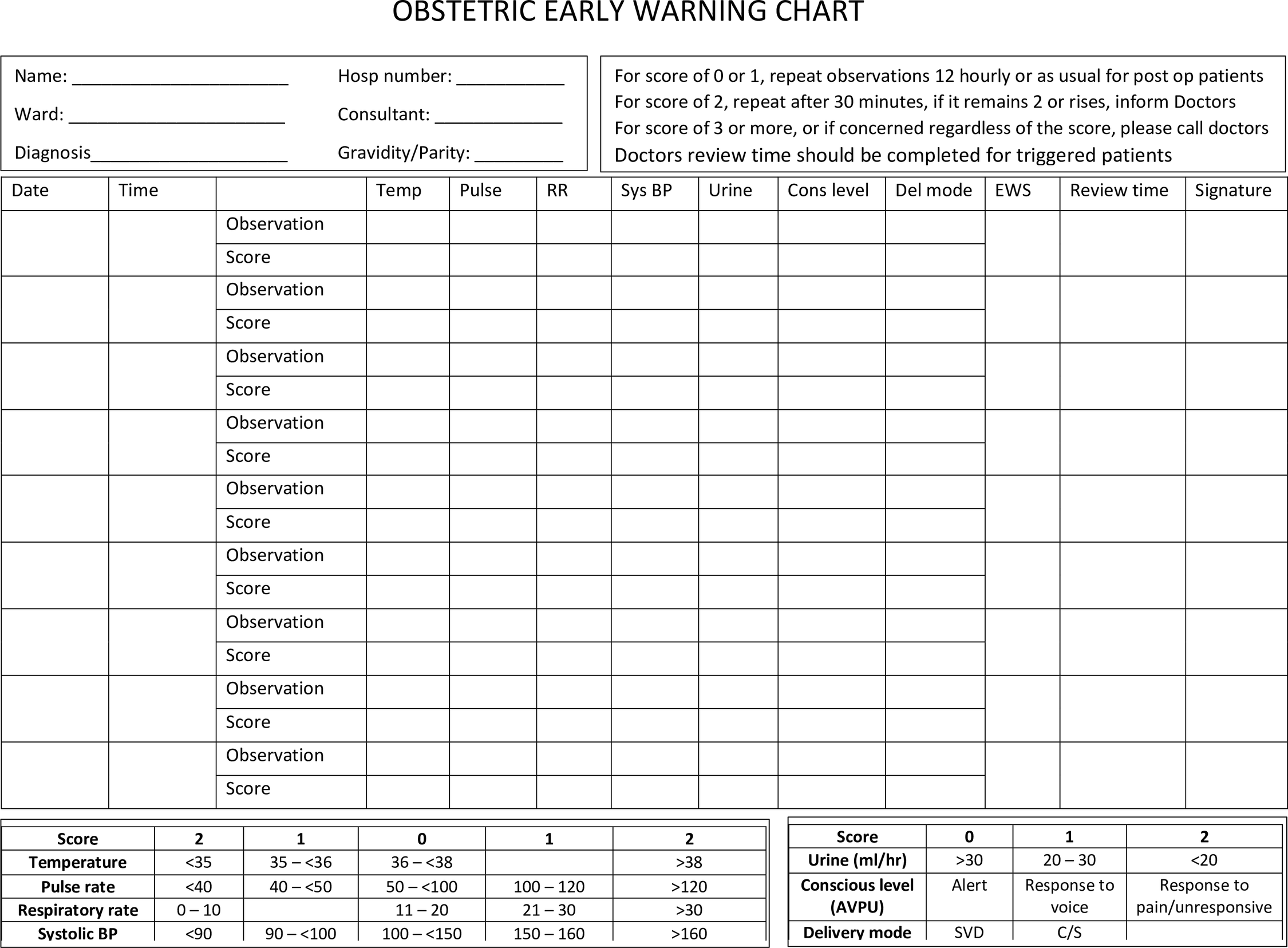

Supplement: S1 Fig — (TIF) [file pgph.0000225.s001.tif]

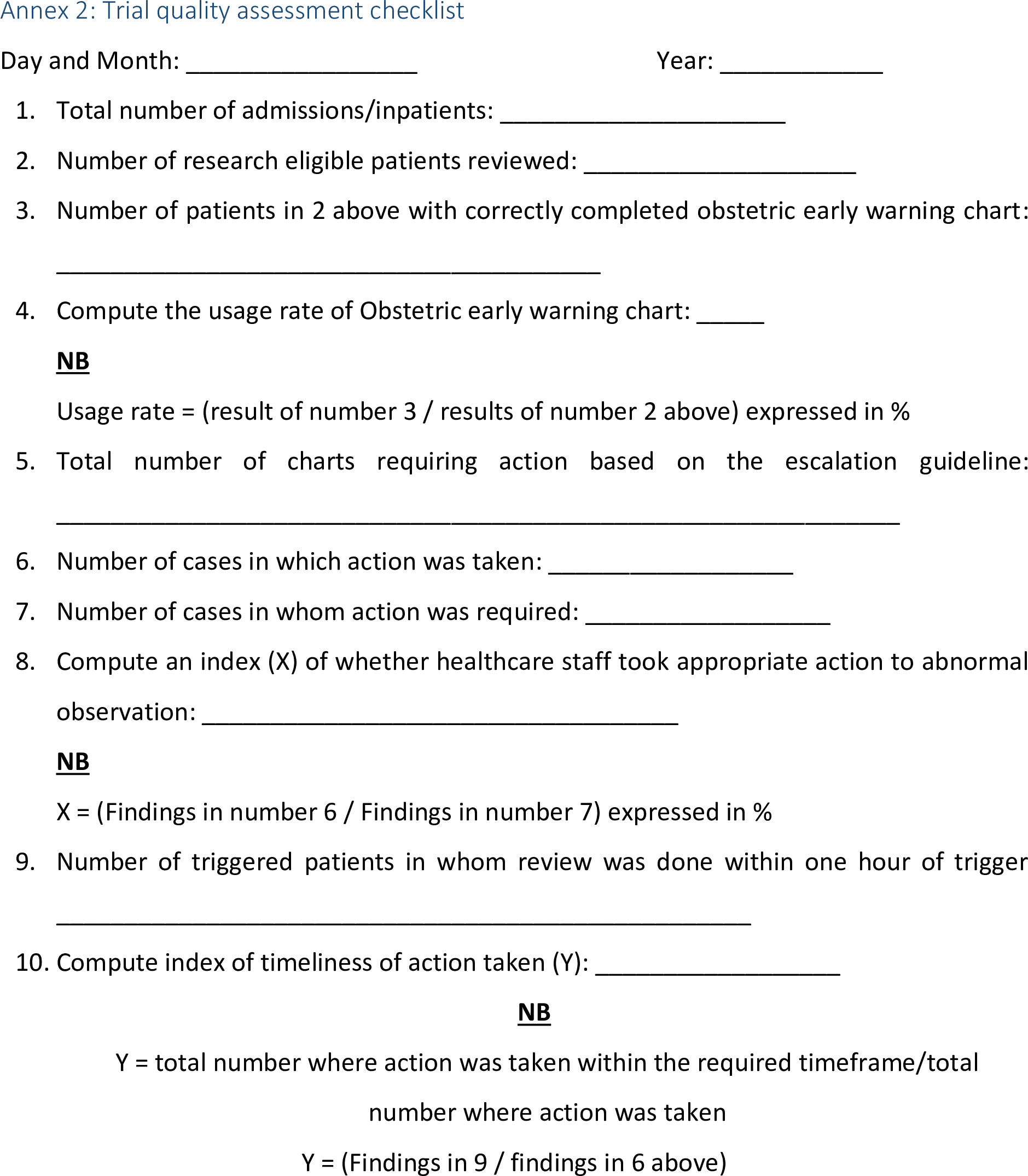

Supplement: S2 Fig — (TIF) [file pgph.0000225.s002.tif]

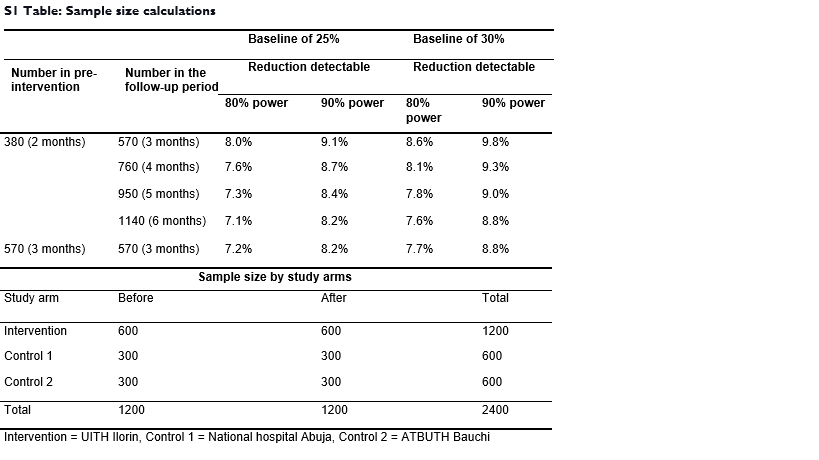

Supplement: S1 Table — (TIF) [file pgph.0000225.s003.tif]

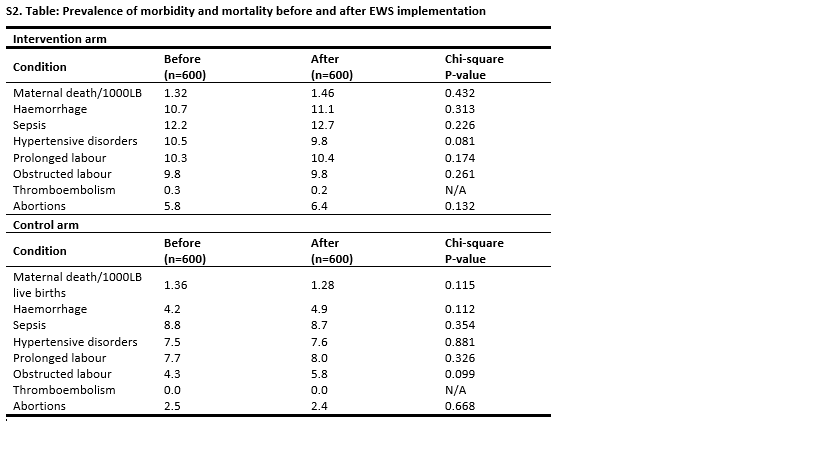

Supplement: S2 Table — (TIF) [file pgph.0000225.s004.tif]
