# Supplementary material for: Correction: Implementation and Evaluation of Obstetric Early Warning Systems in Tertiary care Hospitals
Source: PLOS Glob Public Health. 2023 Apr 4;3(4):e0001807. doi: 10.1371/journal.pgph.0001807 (PMC10072483; doi:10.1371/journal.pgph.0001807)
Supplement: S2 File — (PDF) [file pgph.0001807.s002.pdf]

PLOS GLOBAL PUBLIC HEALTH

RESEARCH ARTICLE

Implementation and evaluation of obstetric early warning systems in tertiary care hospitals in Nigeria

Aminu Umar[1]*, Saidu Ibrahim[2], Idris Liman[3], Calvin Chama[4], Munirdeen Ijaiya[2], Matthews Mathai[5], Charles Ameh[5]

1 Durham and Tees Valley GP Training Program, Health Education North-East of England, Newcastle, United Kingdom, **2** University of Ilorin Teaching Hospital, Ilorin, Nigeria, **3** National Hospital Abuja, Abuja, Nigeria, **4** Abubakar Tafawa Balewa University Teaching Hospital, Bauchi, Nigeria, **5** Centre for Maternal and Newborn Health, Liverpool School of Tropical Medicine, Liverpool, United Kingdom

* amin.umar@nhs.net

Abstract

Obstetric Early Warning Systems (EWS) use combined clinical observations to predict increased risk of deterioration and alert health workers to institute actions likely to improve outcomes. The objective of this study was to explore the experience of health workers about the implementation of an obstetric EWS and assess its effectiveness as an alternative clinical monitoring method compared to standard practice. This mixed-method study included obstetric admissions (n = 2400) to inpatient wards between 01/08/2018 and 31/03/2019 at three Nigerian tertiary hospitals (1 intervention and two control). Outcomes assessed were the efficiency of monitoring and recording vital signs using the patient monitoring index and speed of post-EWS trigger specialist review. These were evaluated through a review of case notes before and four months after EWS was introduced. Qualitative data was collected to explore healthcare workers' views on EWS' acceptability and usability. EWS was correctly used in 51% (n = 307) of the women in the intervention site. Of these women, 58.6% (n = 180) were predicted to have an increased risk of deterioration, and 38.9% (n = 70) were reviewed within 1 hour. There was a significant improvement in the frequency of vital signs recording in the intervention site: observed/expected frequency improved to 0.91 from 0.57, p<0.005, but not in the control sites. Health workers reported that the EWS helped them cope with work demands while making it easier to detect and manage deteriorating patients. Nurses and doctors reported that the EWS was easy to use and that scores consistently correlated with the clinical picture of patients. Identified challenges included rotation of clinical staff, low staffing numbers and reduced availability of monitoring equipment. The implementation of EWS improved the frequency of patient monitoring, but a larger study will be required to explore the effect on health outcomes. The EWS is a feasible and acceptable tool in low-resource settings with implementation modifications.

Trial registration: ISRCTN, ISRCTN15568048. Registration date; 9/09/2020- Retrospectively registered, http://www.isrctn.com/ISRCTN15568048

OPEN ACCESS

Citation: Umar A, Ibrahim S, Liman I, Chama C, Ijaiya M, Mathai M, et al. (2022) Implementation and evaluation of obstetric early warning systems in tertiary care hospitals in Nigeria. PLOS Glob Public Health 2(7): e0000225. https://doi.org/10.1371/journal.pgph.0000225

Editor: Julia Robinson, PLOS: Public Library of Science, UNITED STATES

Received: November 1, 2021

Accepted: June 8, 2022

Published: July 20, 2022

Copyright: © 2022 Umar et al. This is an open access article distributed under the terms of the Creative Commons Attribution License, which permits unrestricted use, distribution, and reproduction in any medium, provided the original author and source are credited.

Data Availability Statement: A record containing information about the dataset set has been put on the LSTM Archive, managed by the LSTM Library. However, the files containing personal information would only be available on request. The files will be stored securely on an LSTM server, which is not accessible to the public and managed by LSTM's central Centre for Data team. Requests to access the full dataset can be made via centrefordata@lstmed.ac.uk.

Funding: This study is part of the doctorate thesis of the first author. The research is fully funded by the Nigerian Petroleum Trust Development Fund (ID 16PhD152). We acknowledge additional funding by Johnson and Johnson's grant (Charity Number 222655) for CEmOC training in Kwara State, Nigeria. The funders had no role in study design, data collection and analysis, decision to publish or preparation of the manuscript.

Competing interests: The authors declare that they have no competing interests.

Introduction

Although the global maternal death burden has fallen by almost 50 per cent in the last two decades, an estimated 810 deaths occur daily around the world due to complications of pregnancy and childbirth [1]. Most of these deaths (94%) occurred in low-resource settings, and most could have been prevented [1]. It is also estimated that there are 27 million episodes of direct obstetric complications annually, which contribute to long-term pregnancy and childbirth complications [2].

Nigeria accounted for 20% of the reported global maternal deaths in 2015 (WHO Factsheet, 2016) [3]. Marked inequalities exist, with northern regions in Nigeria having significantly higher maternal deaths than the southern regions [4].

Increased facility-based births have been reported in the last 15 years, in all WHO regions, with the proportion of births attended by skilled health personnel rising from 56% in 1990 to almost 80% by 2017 [3]. With the resulting increase in the utilisation of health services, a higher proportion of preventable maternal morbidity and mortality has moved from communities to health facilities. Consequently, opportunities to ensure good quality facility care, including timely diagnosis and management of obstetric complications are critical, if the new ambitious maternal health targets of the Sustainable Development Goals (SDG) are to be achieved [4].

Early Warning Systems (EWS) have been developed to facilitate the timely presence of appropriately skilled staff to attend to clinically deteriorating patients [5]. They provide the opportunity to aggregate the impact of sometimes subtle deteriorations in physiological observations into an overall score that, when abnormal, is used to prompt a clinical response [5]. However, the EWS designed for the general population does not account for the unique physiology of pregnant women, and it does not effectively identify at-risk obstetric patients [6].

Obstetric EWS is recommended for monitoring the condition of hospitalised pregnant and postnatal women based on predetermined abnormal values (warning signs) to generate a rapid medical response and facilitate early detection and management of clinical deterioration [5–9]. A recent systematic review of EWS used in obstetrics reported that they are effective in predicting adverse obstetric outcomes and reducing obstetric morbidity [10].

Several EWS have been developed for obstetric patients, but the majority are the result of clinical consensus rather than formal statistical analyses or were created using data from patients admitted to intensive care units, limiting their generalizability to non-intensive care settings [6, 9, 11–15]. Using secondary data on obstetric inpatients admitted to 42 Nigerian tertiary hospitals, Umar et al. (2020) developed and internally validated a simple obstetric diagnostic prediction model and EWS for use in resource-limited settings using recommended methodologies [15–17]. The resulting EWS model performed excellently in predicting Severe Maternal Outcome (SMO: maternal death or near-miss) in the derivation data set with area under the receiver operator characteristic curve (AUROC) consistently above 90% [16]. The objectives of this study were to explore the experience of health workers about the implementation of this EWS and to assess its effectiveness as an alternative clinical monitoring method compared to standard practice.

Materials and methods

Hypothesis

This study tested the hypothesis that the statistically developed and internally validated EWS by Umar et al. (2020) will provide an easier, more convenient and efficient alternative clinical monitoring method than the routine practice [16]. Across all three hospitals, the usual practice

was to record vital signs every 6 hours, or as specified by managing clinicians where more frequent monitoring is required.

Study design

A mixed-method research design using a controlled before-after quasi-experimental trial, qualitative interviews and focus group discussions was employed to achieve the study objectives. The study was conducted over an 8-month period (between 1 August 2018 and 31 March 2019). The choice of study design was based on practical feasibility [18].

Ethics

Ethical approvals were received from the Research and Ethics Committee of the Liverpool School of Tropical Medicine (LSTM- Research Protocol 18–074) and the three study sites (NHA/OG/GC/0171, UITH/CAT/189/19/167, ATBUTH/ ADM /42 / VOL1)**.** Since routinely collected patient information was used for the baseline/pre-implementation phase, no individual-level consent was deemed necessary as was performed in similar studies [19–21]. Participation in the prospective study was voluntary. Written informed consent was obtained for all participants using the modified WHO Research Ethics Committee template for informed consent. This was undertaken by staff nurses and midwives at the point of recruitment (for the trial), a trained research assistant and the principal investigator (For qualitative interviews and FGDs).

Setting

The study was implemented in 3 tertiary care teaching hospitals across regions of northern Nigeria. The EWS was implemented in the 600-bed multispecialty University of Ilorin Teaching Hospital, a public tertiary health care centre located in the north-central zone. The obstetric and gynaecology unit of the hospital has a 208-bed capacity for obstetric and gynaecology cases managed by four teams of consultants, registrars and medical interns. The control sites, purposively sampled were 850-bed capacity National Hospital, Abuja (control 1) and Abubakar Tafawa Balewa University Teaching Hospital, Bauchi (650-bed capacity, control 2). These are located in the north-central and northeast zones, respectively.

Participants and recruitment

Pregnant and postpartum women admitted to inpatient wards due to complications developing antepartum or during the puerperium (42 days postpartum) were included in the study. Women were excluded if they were in active labour, were discharged within 24 hours of normal vaginal birth, or had met any of the three maternal near-miss criteria before hospital admission (clinical, management-based and organ dysfunction-based criteria) [19]. Women who were admitted directly to the intensive care unit without going through any of the inpatient wards were also excluded. Recruitment of participants was conducted by trained nurses and midwives undertaking clinical monitoring in the respective wards.

Intervention

The intervention was the use of a statistically developed obstetric EWS, details of which were published elsewhere [16]. The resulting EWS chart (**S1 Fig**: *EWS chart*) was introduced to replace the vital signs charts of all recruited participants at the intervention site. Briefly, this is a simple score-based recording chart for vital signs. It includes seven clinical parameters (temperature, pulse rate, respiratory rate, systolic blood pressure, diastolic blood pressure,

consciousness level based on the AVPU (alert, voice, pain and unresponsive scale) and mode of birth for postpartum women). Each parameter is scored as 0 for normal, 1 for mild and 2 for severe derangements. An escalation protocol at the top of the chart guides the frequency of patient monitoring and when to trigger clinicians' review (**S1 Fig:** EWS chart); scores of 0 or 1 are reassuring; hence requires 12-hourly monitoring or as routine for post-operative patients. A score of 2 indicates the need to repeat observations after 30 minutes; if the score remains the same or rises, this should 'trigger' doctors' review (doctors should be informed for review). Those with scores of 3 or more are likely to deteriorate clinically and require immediate review. The two control sites were asked to continue with the existing practices of clinical monitoring. This entails using temperature, pulse, blood pressure and respiratory rate (TPR monitoring) charts to record observations. Across all three hospitals, the usual practice was to record vital signs every 6 hours, or as specified by managing clinicians where more frequent monitoring is required.

Outcome measures

Quantitative outcomes assessed were efficiency of monitoring and recording vital signs using the patient monitoring index (PMI), defined as the ratio of the observed to the expected frequency of vital signs monitoring over 24 hours, and speed of post-EWS trigger specialist review measured in minutes.

Sample size calculation

Preliminary data from the intervention site showed that the average monthly obstetric admission was 190 patients. About 3 in 10 of these admissions had direct obstetric complications (haemorrhage, sepsis, abortion complications, hypertensive disorders, prolonged/obstructed labour and thromboembolism). The sample size estimate was made using a baseline prevalence of 25–30% for direct obstetric complications. We considered a three-month period for each of the pre-and post-intervention follow-ups as any further increase does not significantly improve the power (**S1 Table** Sample size calculations). Factoring in a possible exclusion rate of around 5%, the sample size considered was 1200 in the intervention site (600 each pre-and post-intervention). The same numbers of participants (600 each pre-and post-intervention) were to be recruited in the control sites (**S1 Table** Sample size calculations).

Trial procedure

Training workshops on EWS were conducted in the intervention hospital in November 2018. Following this, the hospital management updated the hospital's guidelines for patient monitoring. Specifically, the EWS was to replace routine vital signs charts for all obstetric inpatients (emergency, antenatal, postnatal medical, postnatal surgical and gynaecology wards). A local implementation team of doctors and nurses was constituted to support implementation. They provided on-the-job training on-demand regarding the use of the EWS, including the use of an escalation protocol demonstrated through the use of practical case scenarios. The team trained all staff nurses and midwives undertaking patient monitoring across all obstetric wards. They also responded to any queries about the implementation of the EWS during the postimplementation period. Management of specific complications was as per hospital protocol or usual practice as appropriate.

No incentives were given to nurses/midwives for recruiting women into the research to minimize bias. Compliance was audited by the local implementation team daily until all obstetric inpatients had the EWS in their case notes, at which point recruitment began. Thereafter, weekly audits were conducted to ensure that the charts were correctly used on all

participants. Additionally, formal monthly audits were performed by the principal researcher on a randomly selected date to monitor the use of the chart and any ongoing change in practice. A quality indicator was adapted from that described by Merriel et al. (2017) to provide the implementation team with an easy but objective way to assess the quality of the implementation (S2 Fig: Implementation quality audit checklist) [19–21]. This indicator was used to audit the EWS charts of all obstetric inpatients on the day of the audit. The quality indicator measures three essential ratios: 1) the usage rate of charts (number of patients with correctly completed EWS charts/number of charts reviewed), 2) whether healthcare staff took appropriate action on abnormal observations (number of cases in which action was taken/total number of charts requiring action), and 3) the timeliness of the action if one was required (total number where the action was taken within the required time frame/total number where the action was taken).

Quantitative data collection and management

Baseline retrospective case note reviews of 1200 obstetric admissions to the three health facilities between 1 August and 31 October 2018 was conducted by three research assistants. Android data collection devices (ODK Collect) were used for all quantitative data collection.

Following this, the EWS chart was implemented in the intervention hospital in November 2018. A transition period of two weeks was allowed to audit implementation before the recruitment commenced for prospective post-implementation data. Following this, EWS charts were incorporated into the medical files of recruited participants across all five obstetric inpatient wards. They were prospectively followed until the end of the stay in hospital (discharge or demise), during which a dedicated research assistant (a medical intern) retrieved all completed EWS charts for analysis daily. Women in active labour were excluded and monitored with partograph as defined by the study protocol. Data were collected in the two study arms for four months after implementation (1 December 2018 to 31 March 2019) until the desired sample size (n = 600) was achieved. Post-implementation data was collected through retrospective review of case notes (n = 600) in the two control sites.

These data were exported to a Microsoft Excel spreadsheet and cleaned for analysis. Data analysis was conducted using IBM SPSS version 23. The normality of the distribution of variables was assessed by using distribution plots and Shapiro-Wilk testing. Cumulative and facility-specific characteristics were summarized by mean (SD) for continuous variables and percentages for categorical variables. Descriptive statistics were calculated.

Outcome measures were compared within and between study arms using independent sample t-testing and chi-square for continuous and categorical variables, respectively. $P<0.05$ was considered statistically significant.

Qualitative data collection and management

Semi-structured key informant interviews (KII) and focus group discussions (FGD) were conducted in the intervention hospital at the end of the follow-up period in April 2019 to explore the experience of health workers in the use of the EWS.

The KIIs participants (n = 12) purposively selected were senior midwives/nurses in administrative positions and doctors in the Obstetrics department. The FGDs (n = 6) were conducted with junior nurses/midwives who undertake monitoring of obstetric patients using the EWS. Through the FGD, we aimed to explore and understand their experience of implementing the EWS.

Open-ended interview questions were predesigned based on the study objectives and emerging themes during the post-implementation data collection. Interviews and FGDs were

conducted in English language, led by the principal researcher and research assistants who were not known to the participants. The sessions conducted at the administrative areas of the obstetric department were audio-recorded and transcribed verbatim. Data were collected until saturation and analysed using the thematic framework approach [22].

Patient and public involvement statement

Patients were not involved in the development of the research question or design of this study. Secondary/routinely collected patient monitoring data was used for this analysis. Implications of this study will be disseminated through patient groups and blogs in the study setting.

Results

Recruitment

Overall, 4258 women were admitted to the three hospitals for childbirth or with pregnancy complications between 1 August 2018 and 31 March 2019. A total of 3997 live births and 273 stillbirths were recorded, placing the overall SBR at 63.9/1000 births. Nearly one in five births was preterm (before 37 completed weeks of gestation).

During the baseline pre-implementation period (1 August to 31 October 2018), Women (n = 1200) were recruited into the study retrospectively, comprising 600 in the intervention site and 300 each in the two control sites. Following surveillance, a target of 100% implementation of EWS among all obstetric admissions to five inpatient wards in the intervention site was achieved by the end of November 2018.

Recruitment started from 1 December 2018 in the intervention arm. The highest recruitment rate (95.2%) was reported in December 2018. However, the recruitment rate fell significantly in January 2019 when only 70.8% of research-eligible patients were recruited into the study but this rose steadily thereafter, reaching a peak of 78.1% by the end of March 2019. Overall, the required sample size (n = 600) was achieved after four months (1 December 2018 to 31 March 2019), with an average recruitment rate of 78.8% (Fig 1).

Post-implementation data was collected through a retrospective review of case notes (n = 600) in the two control sites.

Baseline characteristics

The characteristics of the women in the study are illustrated in Table 1. There was no difference in age between the intervention and control groups at baseline (p = 0.348) and post-implementation (p = 0.169). More women were registered for antenatal care in the control hospitals at baseline (p = 0.024); however, this difference was insignificant in the post-implementation cohort (p = 0.155) (Table 1).

Fifty women died due to causes related to pregnancy or childbirth across the three health facilities, putting the cumulative estimated MMR at 1052 per 100,000 live births. Facility-level estimates showed a similar prevalence of maternal death in the intervention site and control hospital-2 (institutional MMR of 1393 and 1320 per 100,000 live births respectively), both having over three times as many deaths as control hospital-1 (institutional MMR of 440 per 100 000 live births).

Maternal morbidity rate was higher in the intervention hospital. During the pre-implementation period, twice as many women suffered an obstetric haemorrhage in the intervention hospital compared to the controls. Similarly, the prevalence of obstructed labour and abortions in the intervention arm was twice that of the control hospitals. The commonest obstetric complication was sepsis, seen in 12.2% and 8.8% of obstetric admissions in intervention and

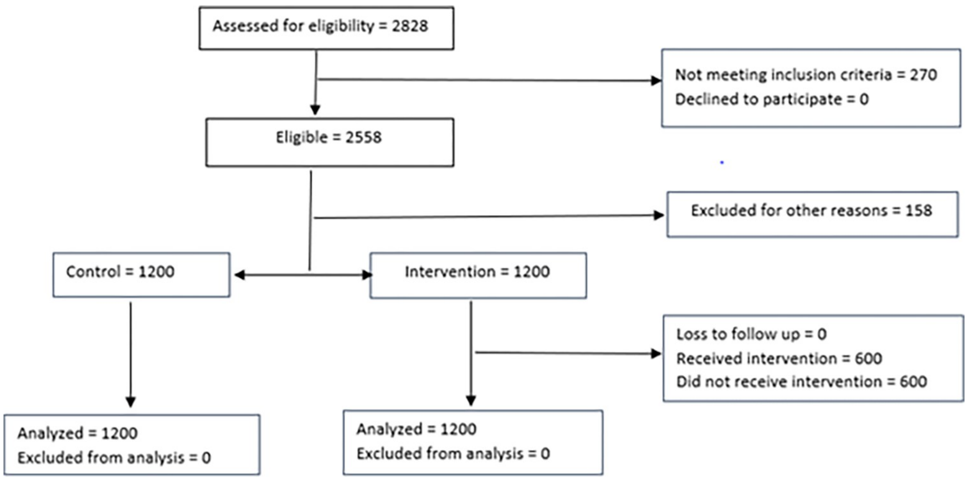

Fig 1. CONSORT flow diagram showing recruitment in intervention site (Dec 2018 –March 2019).

https://doi.org/10.1371/journal.pgph.0000225.g001

control hospitals, respectively. Although the prevalence of both hypertensive disorders and prolonged labour were higher in the intervention site, the difference was not statistically significant, nor was the difference in ICU admission rates (Table 1).

A similar distribution of maternal morbidity was seen across study arms in the post-implementation period (Table 1). Women were nearly three times more likely to suffer obstetric haemorrhage or abortions (16.5%) and twice as likely to have obstructed labour (9.8%) in the intervention hospital compared to the controls (6.7% and 4.3%, respectively). Sepsis also remained the commonest complication, affecting 12.7% and 8.7% of obstetric admissions in the intervention and control hospitals, respectively (Table 1).

Table 1. Characteristics of study participants.

Characteristic	Baseline (n = 1200)			Post-implementation (n = 600)		
	**Intervention** (n = 600)	**Control** (n = 600)	**P-value**	**Intervention** (N = 600)	**Control** (n = 600)	**P-value**
Mean age (SD) (years)	30.0 (5.3)	28 (6.4)	0.348	30 (5.2)	28 (6.3)	0.169
Mean (SD) weight (kg)	72.0 (14.4)	63 (14.2)	**0.038**	70 (11.7)	62.7 (13)	**0.023**
Mean (SD) height (m)	1.6 (0.05)	1.6 (0.1)	0.673	1.6 (0.06)	1.6 (.09)	0.334
Mean (SD) LOS (days)	3.6 (3.2)	2.4 (1.8)	0.368	3.7 (3.5)	2.3 (1.6)	0.714
Booked (%)	60.8	72.2	**0.024**	65.7	68.4	0.155
Booking GA (weeks)	24.8 (8.4)	25.5 (8.1)	0.906	24.6 (8.4)	25.1(8.1)	0.531
ANC visits	2.6 (1.3)	4.3 (2.3)	**0.036**	2.7 (1.7)	4.4 (2.3)	**0.042**
Parity	2.2 (1.4)	2.9 (2.3)	0.305	2.2 (1.3)	3.0 (2.3)	0.181
**Obstetric complications (%)**	**Baseline (n = 1200)**			**Post-implementation (n = 600)**		
	**Intervention** (n = 600)	**Control** (n = 600)	Chi-sq. **P-value**	**Intervention** (N = 600)	**Control** (n = 600)	Chi-sq. **P-value**
Haemorrhage	10.7	4.2	**0.027**	11.1	4.9	**0.019**
Sepsis	12.2	8.8	**0.044**	12.7	8.7	**0.040**
Hypertensive disorders	10.5	7.5	0.125	9.8	7.6	0.331
Prolonged labour	10.3	7.7	0.472	10.4	8.0	0.533
Obstructed labour	9.8	4.3	**0.018**	9.8	5.8	**0.049**
Thromboembolism	0.3	0.0	NA	0.2	0.0	NA
Abortions	5.8	2.5	**0.023**	6.4	2.4	**0.040**

LOS- length of hospital stay. GA- gestational age.

https://doi.org/10.1371/journal.pgph.0000225.t001

Overall, although morbidity prevalence varied across study arms, with the intervention arm having higher rates, there was no change in prevalence within the trial arms following EWS implementation (S2 Table Prevalence of morbidity and mortality before and after EWS implementation).

Completion and trigger rate of EWS

Overall, recording of EWS parameters was incomplete, with regular monitoring (at least twice in 24 hours) of temperature, pulse, respiratory rate and blood pressure performed in 54% of the study participants (Fig 2). Most patients (over 89.2%) had all vital signs monitored and recorded at least once in 24 hours.

Although monitored and recorded, EWS parameters were converted and summed into an EWS score in significantly fewer patients; only 15.4% (n = 92) of the study participants had EWS scores documented as prescribed by the study protocol (at least twice in 24 hours). About half of the study participants (51.2%, n = 307) had EWS scores recorded at least once in 24 hours (Fig 2).

About 58.6% (n = 180) of the women(n = 307)who had EWS scores documented at least once in 24 hours required medical review by a doctor (Fig 2). Of these, 38.9% (n = 70) were reviewed by a doctor. In terms of timeliness of the review, about three-quarters of the reviewed patients (75.7%, n = 53) had the time of doctor's review correctly documented on the EWS chart; all of these patients were reviewed within 60 minutes, as recommended by the EWS escalation protocol.

The frequency of monitoring of patients was assessed using PMI for the four routinely monitored vital signs (respiratory rate, temperature, pulse rate and blood pressure). Across all three hospitals, the guidelines for monitoring obstetric patients using the vital signs chart is to monitor them every 6 hours (at least four times in 24 hours). While this applied for the intervention hospital during the baseline period, the expected frequency of monitoring during the post-implementation period was as specified by the EWS escalation protocol; i.e. twice daily for EWS scores of 0 or 1, 30-minutes apart for a score of 2 and immediate referral for scores of 3 or more.

Significant improvement in the frequency of monitoring was observed in the intervention hospital (Table 2). This was especially so for temperature and respiratory rate monitoring,

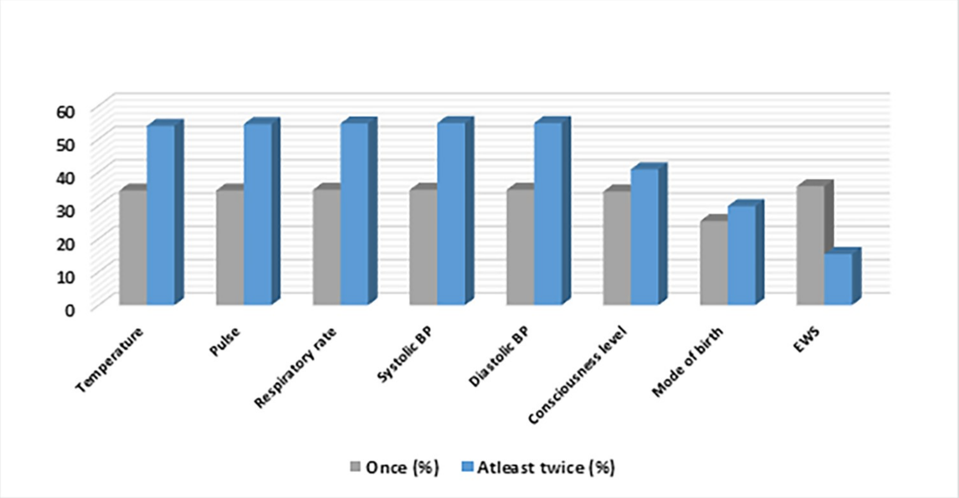

Fig 2. Completion rate of the EWS parameters and trigger system.

https://doi.org/10.1371/journal.pgph.0000225.g002

Table 2. Frequency of vital signs monitoring before and after EWS implementation.

Intervention Hospital: Patient Monitoring Indices (PMI)			
	**Baseline**	**After**	**t-test (p)**
Temp (SD)	0.5 (0.4)	0.9 (0.4)	<0.005
Pulse (SD)	0.6 (0.3)	0.9 (0.4)	<0.005
RR (SD)	0.5 (0.4)	0.9 (0.4)	<0.005
BP (SD)	0.6 (0.3)	0.9 (0.4)	<0.005
Control Hospitals: Absolute monitoring frequencies			
	**Baseline**	**After**	**t-test (p)**
Temp (SD)	1.7 (0.9)	1.6 (0.9)	0.234
Pulse (SD)	1.8 (0.9)	1.7 (0.9)	0.123
RR (SD)	1.8 (1.3)	1.8 (1.2)	0.221
BP (SD)	1.8 (0.9)	1.7 (0.9)	0.115

https://doi.org/10.1371/journal.pgph.0000225.t002

with baseline mean (SD) PMI of 0.5 (0.4) and 0.5 (0.4), and post-implementation mean (SD) PMI of 0.9 (0.4) and 0.9 (0.4), respectively. No significant change in the frequency of vital signs monitoring was observed in both control hospitals (**Table 2**).

Experience and challenges of using EWS

Twelve KIIs and FGDs were conducted with the clinical staff in the intervention site. Experience of using EWS and challenges encountered during the study period were explored among participants using open-ended interview questions and prompts. Interviewees consisted of seven senior nursing officers (five chief nursing officers heading the five wards: gynae emergency, antenatal, postnatal medical, postnatal surgical and gynaecology wards, and two assistant chief nursing officers) and five medical doctors (two senior registrars, two registrars and one intern in the Obstetrics and Gynaecology department). FGDs were conducted with nurses and midwives, each session having five participants from the participating wards.

Most of the nurses/midwives found the EWS chart useful in alerting them when to escalate care to doctors. They reported that abnormal observations are usually an indicator that the patient needs more frequent monitoring. In addition to contributing to the early detection of deterioration, they felt the chart assisted them directly in managing sick patients [Box 1].

Box 1. Staff comments on the EWS

- *Clinical information of patients is compressed into a single score, making it easy to evaluate at a glance . . . (FGD nurse)*
- *It was accurate in that all patients with high scores are always the sick ones. In fact, it even assists us in monitoring how our post-operative patients are recovering after a caesarean section. . . (KII Nurse)*
- *You know interns rotate, so are nurses; most of the errors in scoring are caused by lack of continuous training (KII Doctor)*
- *we don't have enough staff, like in the postnatal medical ward, you find only one nurse covering most afternoon shifts. . . (KII nurse)*

- *like here, we have only one BP machine on the ward, and to check a patient's oxygen saturation, you have to transfer her to the gynaecology emergency ward. . . (FGD nurse)*
- *A coloured chart would be easier to use than this. . . I am sure you chose black and white because of low production cost, but the management should adopt the coloured chart. . . (KII Doctor)*

Compared to the routinely used vital signs chart, most of the nurses felt EWS was easier to use because of less frequent monitoring of clinically stable patients. By scoring vital signs and having a cumulative EWS score, the chart "compresses clinically relevant parameters into a simple score, making it easy to evaluate patients at a glance (KII nurse)".

The doctors opined that EWS was a good monitoring tool if properly followed. They found the charts easy to correlate with a patient's clinical picture, with abnormal scores usually consistent with clinical deterioration. They also felt the chart could potentially help nurses to cope with the demands of their work, given the gross shortage of human resources for health, while making it easier to detect unwell patients [Box 1].

Overall, most interviewees agreed a colour-coded EWS would be easier to use and more efficient in picking out and communicating the need for clinical review. Additionally, it would be less labour-intensive and more visually appealing, hence more likely to be accepted by clinical staff.

Major limiting factors to effective monitoring of vital signs using EWS were the shortage of functioning equipment and frequent staff rotation by the hospital management [Box 1]. There was a gross shortage of patient monitoring equipment across all five wards. Although the hospital management had approved the use of the EWS instead of the routinely used vital signs charts, some nurses reported having to use the old monitoring chart concurrently with the EWS charts, potentially increasing the workload of staff and stretching the use of scarce patient monitoring equipment.

Rotations of nurses/midwives (and medical interns), which happen every 6-months, brings in new clinical staff who are untrained in the use of EWS [Box 1]. This happened shortly after the EWS implementation, taking most of the trained nurses to other clinical departments. This significantly affected the recruitment rate and overall success of the study. A few midwives reported that the escalation protocol was ambiguous, hence a common cause of error in patient monitoring, especially among newly deployed staff nurses. The training provided was said to be grossly inadequate.

Discussion

The patient monitoring index showed a significant improvement following EWS implementation in the intervention hospital. This was especially so for temperature and respiratory rate but also significant for the other EWS parameters. With a similar baseline frequency of vital signs monitoring as the intervention hospital, no change in frequency was observed in the control hospitals during the post-implementation period. This is consistent with findings of one before-after study, which reported an increase in the frequency of documentation of vital signs following the implementation of the Irish Maternity EWS on obstetric patients with sepsis [23]. Our findings are consistent with other studies that reported improvement in the post-operative monitoring of women after caesarean section following the introduction of modified obstetric EWS [20, 21, 24, 25].

The average institutional maternal mortality ratio (MMR) across the 3 study sites was 1052 per 100,000 live births. This is significantly higher than the population-based national average of 917 per 100,000 live births [1] but comparable to the estimated institutional MMR from Nigerian tertiary health facilities [26]. Maternal mortality reviews indicate that a significant proportion of women who die due to pregnancy and childbirth demonstrate abnormal vital signs long before death, suggesting that a multi-parameter EWS system should identify them with high specificity [6, 17, 27]. Effectively, this should facilitate timely diagnosis and treatment, limit the severity of morbidity, and possibly reduce mortality. Obstetric EWS have been previously shown to prevent progressive obstetric morbidity [14, 28–30]. Shields and colleagues reported a significant reduction in severe and composite maternal morbidity ($p<0.01$) as defined by the Centre for Disease Control (CDC), but not mortality, in six intervention hospitals following EWS implementation, compared to 19 controls [30]. However, they observed no change in the ICU admission rate in either the intervention or control hospitals [30]. Maternal mortality and near-miss are rare outcomes. Therefore a considerably large sample size is required to have a substantial number for effective analysis. Given that the implementation of EWS in this study involved a single facility, a large multicentre randomized controlled trial will be more appropriate to evaluate the effectiveness of obstetric EWS in reducing severe obstetric complications and death.

Caesarean births constituted 31.8% of all births in the three hospitals. This is a significant increase from a 2009 report of hospital-level CS rate of 14% in Nigerian tertiary hospitals [31]. In the last three decades, the caesarean section rate has continued to increase in an unprecedented manner in both developed and developing countries, above the WHO-recommended 10% at the population level [3, 31, 32, 33]. This rise is driven by major increases in non-medically indicated CS [32–38]. Following the implementation of EWS, the CS rate dropped significantly in the intervention but not in any of the control facilities. However, it was difficult to attribute this to a reduction in medically unnecessary CS as an analysis of indications for caesarean births during the baseline and post-implementation periods was not performed. This can be explored in future research.

The opinion of clinical staff regarding EWS was generally positive, with the feeling that it helped them to cope with the demands of their work while making it easier to detect and manage deteriorating patients. While these reports are hard to correlate with our observed low usage rate, it is consistent with reports of incomplete recording of clinical parameters in obstetric EWS, especially respiratory rate [19, 21, 24, 25], including where EWS was implemented in well-resourced settings [6, 12, 39].

Comparatively, a low usage rate of a similar monitoring tool, the partograph has been widely reported in Nigeria [40] and other similar settings [41]. Being a major change to the organizational norm, the explanation of our findings may not be farfetched. Whilst many organizations appreciate the need for change, as many as 70% of the change programs have been shown to not achieve their intended outcomes [42]. Fundamental to the success of organisational change is the acceptance of the change by employees (the nurses and midwives) following a period of denial [43]. Within this context, information on the established usefulness of obstetric EWS as screening tools for morbidity and their effectiveness in improving health outcomes was provided to clinical staff early to help tackle their denial [44]. It is essential to check that employees have successfully transitioned through denial to acceptance before full implementation [45]. Although a multi-disciplinary local implementation team was constituted to provide surveillance and support for full implementation, it is likely that persistent denial played a role in our observed low uptake rate. The situation was compounded by the extent of staff transfers observed, with newly deployed staff not trained on the EWS

implementation protocol. Hence, a longer study period with active support from the implementation team could have improved the uptake of the obstetric EWS.

Organizational change involves a three-stage process: Unfreezing current behaviour; then, moving to the new behaviour; and, finally, refreezing the new behaviour [45]. The three-step model was adopted for many years as the dominant framework for understanding the process of organizational change [46]. To put in context, we presented the findings of our feasibility study and the systematic review during the pre-implementation training sessions in the intervention hospital as an evidence base to unfreeze the current practice of patient monitoring. We then trained staff on the use of EWS and provided a mechanism to support and supervise the implementation (local multi-disciplinary implementation team) of the new behaviour (EWS implementation in this case). At the end of our study, we disseminated our findings to the intervention hospital to enhance refreezing of this new behaviour [46, 47].

Although a lot of work has taken place to assess the benefits of obstetric EWS across the globe, to the best of our knowledge, this is the first report of the implementation of an EWS in a low-resource tertiary hospital that was developed and validated using data from low resource settings.

The success of this study is undoubtedly attributable to enormous support from hospital management. Through the approval of EWS being substituted for routine vital signs charts and the dispatching of internal circulars to that effect across all obstetric wards, the major institutional barriers to implementation were broken. Additionally, the fact that the implementation was led by the local multi-disciplinary implementation team under the supervision of the local co-PI, who is a senior professor in the Obstetrics and Gynaecology department, facilitated uptake and ownership of the obstetric EWS.

This study has some limitations. All health facilities included were tertiary hospitals that provided comprehensive emergency obstetric care services. The scope and budget allowed us to have only one hospital in the intervention arm. However, this is a large university teaching hospital servicing a state with a population of 2.37 million [48]. The feasibility and utility of implementing the EWS chart in smaller centres, including primary healthcare facilities, with smaller staff numbers were not investigated in this study. To improve the generalisability of findings, further multicentre studies with multiple intervention sites and across different levels of care (including primary and secondary care hospitals) are needed.

With the before-after design, it is likely that the impact of the EWS implementation on health outcomes will be stronger soon after the intervention has been implemented and that this will reduce with time. This is because the staff (nurses, midwives and interns) trained in each ward may not be retained within the Obstetrics department, mainly due to staff rotation. Once a critical mass of trained staff is lost from the research wards, the use of EWS will likely reduce. Although we employed surveillance and continuous training/retraining of clinical staff, low retention of trained staff remained a major limitation that significantly affected overall compliance with the study protocol.

Conclusion

Findings from this research showed that the obstetric EWS could improve the quality of patient care through better monitoring frequency and medical review based on abnormally high EWS scores. Effective monitoring and timely actions are likely to reduce the risk of mortality. The implementation was not without challenges; however, with staff education on the usefulness of EWS, provision of adequate patient monitoring equipment, coupled with continuous training and retraining of staff, EWS would potentially provide a convenient and efficient alternative patient monitoring method to cope with the unique demands on obstetric practice

in low-resource tertiary healthcare settings. A larger study will be required to explore the effect on health outcomes and to investigate the feasibility and acceptability at lower levels of care.

Supporting information

S1 Fig. EWS chart.
(TIF)

S2 Fig. Implementation audit quality checklist.
(TIF)

S1 Table. Sample size calculations.
(TIF)

S2 Table. Prevalence of morbidity and mortality before and after EWS implementation.
(TIF)

Acknowledgments

We acknowledge the support of our colleagues, Sarah White, Helen Allot, Alexander Manu and Hauwa Mohammed, at the Centre for Maternal and Newborn Health, Liverpool School of Tropical Medicine.

Author Contributions

Conceptualization: Aminu Umar, Charles Ameh.

Data curation: Aminu Umar.

Formal analysis: Aminu Umar.

Funding acquisition: Aminu Umar.

Investigation: Aminu Umar.

Methodology: Aminu Umar.

Project administration: Aminu Umar, Saidu Ibrahim, Idris Liman, Calvin Chama, Munirdeen Ijaiya, Matthews Mathai, Charles Ameh.

Resources: Aminu Umar.

Supervision: Matthews Mathai, Charles Ameh.

Writing – original draft: Aminu Umar.

Writing – review & editing: Aminu Umar, Saidu Ibrahim, Idris Liman, Calvin Chama, Munirdeen Ijaiya, Matthews Mathai, Charles Ameh.

References

1. WHO, U., (2019). UNFPA, World Bank Group, and the United Nations Population Division. Trends in Maternal Mortality: 2000 to 2017. Geneva. World Health Organization.
2. Machiyama K., Hirose A., Cresswell J., Barreix M., Chou D., Kostanjsek N., et al. (2017). Consequences of maternal morbidity on health-related functioning: A systematic scoping review. BMJ Open, 7(6), 1–12. https://doi.org/10.1136/bmjopen-2016-013903 PMID: 28667198
3. WHO. (2016). Global strategy on human resources for health: workforce 2030.
4. Meh C., Thind A., Ryan B. and Terry A., 2019. Levels and determinants of maternal mortality in northern and southern Nigeria. BMC pregnancy and childbirth, 19(1), p.417. https://doi.org/10.1186/s12884-019-2471-8 PMID: 31718572

5. Morgan R., Williams F., Wright M. (1997). An Early Warning Scoring system for detecting developing critical illness. Clin Intensive Care, 8(82), 100.
6. Lappen J., Keene M., Lore M., Grobman W., Gossett D. (2010). Existing models fail to predict sepsis in an obstetric population with intrauterine infection. American Journal of Obstetrics and Gynecology, 203 (6), 573.e1–5. https://doi.org/10.1016/j.ajog.2010.07.040
7. United Nations. (2018). Goal 3.:. Sustainable Development Knowledge Platform.
8. United Nations. (2019). United Nations. Time for global action for people and planet: Sustainable Development Goals.
9. Alam N., Hobbelink E., Van Tienhoven A. Van de Ven P., Jansma E., Nanayakkara P. (2014). The impact of the use of the Early Warning Score (EWS) on patient outcomes: A systematic review. Resuscitation. 85, pp.587–94. https://doi.org/10.1016/j.resuscitation.2014.01.013 PMID: 24467882
10. ACOG. (2013). American College of Obstetricians and Gynecologists, Committee on Obstetric Practice Safety for Maternal-Fetal Medicine. Committee Opinion No. 573: magnesium sulfate use in obstetrics. 727–728.
11. CEMACH. (2007). The Seventh Report of the Confidential Enquiries into Maternal Deaths in the United Kingdom.
12. Edwards S., Grobman W., Lappen J., Winter C., Fox R., Lenguerrand E., et al. (2015). Modified obstetric early warning scoring performance for severe sepsis in women with chorioamnionitis. The American Journal of Obstetrics & Gynecology, 212(4), 536.e1–536.e8. https://doi.org/10.1016/j.ajog.2014.11.007 PMID: 25446705
13. Mhyre J., D'Oria R., Hameed A., Lappen J., Holley S., Hunter S. et al. (2014). The maternal early warning criteria: a proposal from the national partnership for maternal safety. Obstetrics and Gynecology, 124(4), 782–786. https://doi.org/10.1097/AOG.0000000000000480 PMID: 25198266
14. Umar A, Ameh C, Muriithi F. Matthai M. (2019). Early Warning Systems in Obstetrics: a systematic review of literature. PLOS ONE. 14(5), p.e0217864. https://doi.org/10.1371/journal.pone.0217864 PMID: 31150513
15. Collins G., Reitsma J., Altman D, Moons K. (2015). Transparent Reporting of a multivariable prediction model for Individual Prognosis Or Diagnosis (TRIPOD): The TRIPOD Statement. Annals of Internal Medicine, 162(1), 55. https://doi.org/10.7326/M14-0697 PMID: 25560714
16. Umar A, Manu A, Mathai M. Ameh C. (2020). Development and Validation of Obstetric Early Warning System model for use in low resource settings. BMC Pregnancy and Childbirth. 20(1), pp.1–9. https://doi.org/10.1186/s12884-020-03215-0 PMID: 32917151
17. Carle C., Alexander P., Columb M., Johal J. (2013). Design and internal validation of an obstetric early warning score: secondary analysis of the Intensive Care National Audit and Research Centre Case Mix Programme Database. Anaesthesia, 68(4), 354–367. https://doi.org/10.1111/anae.12180 PMID: 23488833
18. Rogers J; Révész A; (2020) Experimental and quasi-experimental designs. In: McKinley J and Rose H, (eds.) The Routledge Handbook of Research Methods in Applied Linguistics. (pp. 133–143). Routledge: London, UK.
19. Merriel A., Murove B. T., Merriel S., Sibanda T., Moyo S., & Crofts J. (2017). Implementation of a modified obstetric early warning system to improve the quality of obstetric care in Zimbabwe. International Journal of Gynaecology and Obstetrics: The Official Organ of the International Federation of Gynaecology and Obstetrics, 136(2), 175–179. https://doi.org/10.1002/ijgo.12028 PMID: 28099733
20. Sheikh S., Qureshi R., Nausheen, and Sultanali R. (2017). Audit Implementation of warning tool to improve maternal newborn health outcomes. Journal of the Pakistan Medical Association: JPMA 67, no. 1 (2017): 111
21. Singh A., Guleria K., Vaid N, and Jain S. (2016). European Journal of Obstetrics & Gynecology and Reproductive Biology Evaluation of maternal early obstetric warning system (MEOWS chart) as a predictor of obstetric morbidity : a prospective observational study. European Journal of Obstetrics and Gynecology, 207, 11–17. https://doi.org/10.1016/j.ejogrb.2016.09.014
22. Ritchie J., Spencer L. and O'Connor W., (2003). Carrying out qualitative analysis. Qualitative research practice: A guide for social science students and researchers, pp.219–62.
23. Maguire P., Higgins A., Power K. Daly N. McKeating A and Michael J. (2015). Maternal bacteremia and the Irish maternity early warning system. International Journal of Gynecology and Obstetrics, 20–23. https://doi.org/10.1016/j.ijgo.2014.11.022
24. Moore J., Thomson D., Pimentil I., Fekad B. and Graham W. (2019). Introduction of a modified obstetric early warning system (MOEWS) at an Ethiopian referral hospital: a feasibility assessment. BMJ Open Quality, 8(1), e000503. https://doi.org/10.1136/bmjoq-2018-000503 PMID: 31206042

25. Singh S., McGlennan A., England A. Simons R. (2012). A validation study of the CEMACH recommended modified early obstetric warning system (MEOWS). *Anaesthesia*, 67(1), pp.12–18. https://doi.org/10.1111/j.1365-2044.2011.06896.x PMID: 22066604

26. Oladapo O.T., Vogel J.P., Hunter E.C. (2015). The mistreatment of women during childbirth in health facilities globally: a mixed-methods systematic review. PLoS medicine, 12(6). https://doi.org/10.1371/journal.pmed.1001847 PMID: 26126110

27. Paternina-caicedo A., Miranda J., Bourjeily G., Levinson A. Duennas A., Bello- Munoz, et al (2017). Performance of the Obstetric Early Warning Score critically ill patients for the prediction of maternal death. The American Journal of Obstetrics & Gynecology, 216(1), 58.e1–58.e8. https://doi.org/10.1016/j.ajog.2016.09.103 PMID: 27751799

28. Hedriana H., Wiesner S., Downs B., Pelletreau B. and Shields L. (2016). International Journal of Gynecology and Obstetrics Baseline assessment of a hospital-specific early warning trigger system for reducing maternal morbidity. International Journal of Gynecology & Obstetrics, 132(3), pp.337–341.

29. Isaacs R., Wee M., Bick D., Beake S., Sheppard Z., Thomas S. et al. (2014). A national survey of obstetric early warning systems in the United Kingdom: Five years on. Anaesthesia, 69(7), 687–692. https://doi.org/10.1111/anae.12708 PMID: 24801160

30. Shields L., Wiesner S., Klein C., Pelletreau B. and Hedriana H. (2016). Use of maternal early warning trigger tool reduces maternal morbidity. American journal of obstetrics and gynecology, 214 (4), pp.527–e1. https://doi.org/10.1016/j.ajog.2016.01.154 PMID: 26924745

31. Boerma T., Ronsmans C., Melesse D., Aluisio J., Fernando C., Liang J. et al (2018). Global epidemiology of use of and disparities in caesarean sections. The Lancet, 392(10155), 1341–1348. https://doi.org/10.1016/S0140-6736(18)31928-7 PMID: 30322584

32. Vela M., Souza P., & Zhang J. (2014). Searching for the Optimal Rate of Medically Necessary Cesarean Delivery. Birth, 41(3), 237–244 https://doi.org/10.1111/birt.12104 PMID: 24720614

33. Ameh C. Weeks, A. (2009). The role of instrumental vaginal delivery in low resource settings. BJOG: An International Journal of Obstetrics and Gynaecology, 116(SUPPL. 1), 22–25. https://doi.org/10.1111/j.1471-0528.2009.02331.x PMID: 19740166

34. Shah A., Fawole B., M'Imunya, Faouzi A., Nafiou Idi, Jean- Jose W. et al (2009). Cesarean delivery outcomes from the WHO global survey on maternal and perinatal health in Africa. International Journal of Gynecology and Obstetrics, 107(3), 191–197. https://doi.org/10.1016/j.ijgo.2009.08.013 PMID: 19782977

35. WHO 2015. (2015b). WHO Statement on caesarean section rates. Reproductive Health Matters, 23 (45), 149–150. https://doi.org/10.1016/j.rhm.2015.07.007 PMID: 26278843

36. Betran A, Wojdyla D, Posner SF, and Gulmezoglu, A., (2015) National estimates for maternal mortality: an analysis based on the WHO systematic review of maternal mortality and morbidity. BMC Public Health 5: 131 https://doi.org/10.1186/1471-2458-5-131

37. Tunçalp Ö, Were W., MacLennan C., Oladapo O., Gulmezoglu A., Bahl R. et al (2015). Quality of care for pregnant women and newborns-the WHO vision. BJOG: An International Journal of Obstetrics & Gynaecology, 122(8), 1045–1049. https://doi.org/10.1111/1471-0528.13451 PMID: 25929823

38. Okeke T., & Ekwuazi K. (2013). Is there still a place for vacuum extraction (ventouse) in modern obstetric practice in Nigeria. Annals of Medical and Health Sciences Research, 3(4), 471. https://doi.org/10.4103/2141-9248.122043 PMID: 24379994

39. Maguire P., Power K., Daly N., Farren M., McKeating A., & Turner M. J. (2016). Brief communication: High dependency unit admissions during the first year of a national obstetric early warning system. International Journal of Gynecology and Obstetrics, 133, 121–122. Retrieved from https://doi.org/10.0.3.248/j.ijgo.2015.09.014

40. Opiah M, Ofi A, Essien E and Monjok E. (2012). Knowledge and utilization of the partograph among midwives in the Niger Delta Region of Nigeria. Afr J Reprod Health. 2012; 16(1):125–132. PMID: 22783676

41. Mabasa, S, (2018). Evaluation of partogram utilization in maternity care in selected health care facilities of Bojanala District (Doctoral dissertation, North-West University).

42. Balogun J, Hope-Hailey V, Johnson G (ed.) & Scholes K(ed.) (2008), Exploring Strategic Change (3rd ed.). Prentice-Hall, London, U. K.

43. KÜB0LER-ROSS E. (1973). On death and dying. London, Routledge. http://site.ebrary.com/id/10094212.

44. Todnem R. (2005). Organizational change Management: 115. A critical Review//Journal of change Management. 115(5):4.

45. Hussain ST, Lei S, Akram T, Haider M, Hussain SH, and Ali M(2008). Change model: A critical review of the role of leadership and employee involvement in organizational change. Journal of Innovation & Knowledge. 3(3):123–7.

46. Bakari H, Hunjra AI, Niazi GS (2017). How does authentic leadership influence planned organizational change? The role of employees' perceptions: Integration of theory of planned behavior and Lewin's three step model. Journal of Change Management. 17(2):155–87.

47. Fernandez S, Rainey HG. (2006). Managing successful organizational change in the public sector. Public administration review. 66(2):168–76.

48. Dildy G., Belfort M., & Clark S. (2016). Obstetric Forceps: A Species on the Brink of Extinction. Obstetrics and Gynecology, 128(3), 436–439. https://doi.org/10.1097/AOG.0000000000001557 PMID: 27500345
